# Inclusion of phase III clinical trial costs in health economic evaluations

**DOI:** 10.1186/s12913-024-11638-0

**Published:** 2024-10-01

**Authors:** Afschin Gandjour

**Affiliations:** Frankfurt School of Finance & Management, Adickesallee 32-34, Frankfurt am Main, 60322 Germany

**Keywords:** Phase III clinical trial, Health-economic evaluations, Cost-effectiveness, Study protocol, Modeling

## Abstract

**Introduction:**

Protocol-driven trial activities contribute to the utility gain demonstrated in the phase III clinical trial of a new drug. If this utility gain cannot be distinguished from the effects of the new drug itself, protocol-driven trial costs cannot be easily dismissed for consistency reasons. This study aims to estimate the impact of including per-patient costs of phase III clinical trials on the incremental cost-effectiveness ratio (ICER).

**Methods:**

The analysis utilized a modeling approach with secondary data from an ad-hoc literature review, considering both societal and payer perspectives. While the costs of phase III clinical trials may cancel out during the period of “normal” life-years due to the incremental cost calculation, they do not cancel out when differential early treatment termination occurs (e.g., due to differential mortality). Assuming the presence of differential mortality, per-patient phase III trial costs were calculated for the period of added life-years. These costs were then included in the ICER of a new drug, under the assumption that direct patient-related costs constitute 30–70% of the total trial costs. Capital costs were also incorporated from a societal perspective.

**Results:**

Based on assumptions of $40,000 out-of-pocket expenses per patient enrolled in a phase III trial and a life expectancy gain of three months, incremental costs increased by $27,000 from a societal perspective. From a payer perspective, the estimate was $12,000.

**Conclusions:**

The costs of phase III trials are a relevant component of the ICER, and excluding it is generally not appropriate for consistency reasons. Properly considering these trial costs is essential for a comprehensive evaluation of a new drug’s cost-effectiveness.

## Introduction

Phase III clinical trials typically compare a new therapeutic entity (i.e., a new chemical or biological drug) to the standard of care and aim to confirm its efficacy. These trials usually enroll several hundred patients [7, 13, 14]. Phase III clinical trials are the primary drivers of research and development (R&D) costs [4, 12, 17]. However, there is substantial variation in published estimates of the costs of phase III trials. For example, Moore et al. [14] and EvaluatePharma [7] report median costs of US-$19 and US-$127 million, respectively. The costs of phase III trials primarily involve administrative staff (20%), clinical procedures (20%), clinical staff (15%), site monitoring (14%), site retention (11%), and central laboratory (7%) [17, 18].

Protocol-driven costs and activities contribute to the costs of phase III clinical trials and can be allocated to the various cost categories. These costs are necessary to complete the protocol treatment but are typically not incurred outside of the trial, in the real world [8]. Only purely pragmatic trials do not require consideration of protocol-driven costs because they are not incurred.

Protocol-driven activities can be categorized into the following components: (i) activities to enhance patient adherence to medication, (ii) activities for the diagnosis and treatment of conditions that would have remained undetected in clinical practice, (iii) activities for extra testing and data collection, and (iv) activities for quality assurance [9]. Since these protocol-driven activities impact health outcomes and patient utility, they cannot be excluded from a cost-effectiveness analysis (CEA) conducted alongside a clinical trial for the sake of consistency [6, 8]. Despite this, anecdotal evidence suggests that many authors implicitly exclude protocol-driven costs by not addressing them. Authors may also intentionally exclude them, assuming that they are the same in both arms and therefore cancel out, or assuming that they are not incurred in clinical practice. The current practice of excluding protocol-driven costs may also be influenced by the desire to keep the analysis simple or, in cases of conflicts of interest, to demonstrate a favorable incremental cost-effectiveness ratio (ICER). Additionally, current practices may be influenced by the recommendations of international guidelines on economic evaluations. The few guidelines that have explicitly addressed protocol-driven costs either suggest categorical exclusion [3] or exclusion if they do not appear to reflect clinical practice or the target population [2].

Among the various protocol-driven cost components, only the costs to improve patient adherence may be excludable under certain conditions, for example, when it seems appropriate to dichotomize the clinical outcome based on the degree of adherence [8]. As a general rule, all other protocol-driven costs need to be included because the cost and utility impact of the underlying protocol-driven activities cannot be easily separated [9]. The cost and utility impact of protocol-driven activities only cancel out from the ICER under specific conditions (see Methods).

This study aims to determine the change in the ICER when including the costs of protocol-driven expenses. Using a de novo cost-effectiveness model and publicly available data on clinical trial costs and activities, the analysis will be undertaken from both societal and payer perspectives.

## Methods

### Background

In the following section, I will refer to trials with two arms for simplicity. However, it is important to note that the considerations also apply to trials with more than two arms. Furthermore, the methodological approach is not limited to phase III trials but is also applicable to pivotal phase I and II trials.

While costs and utility from protocol activities may be the same in both arms and cancel out in the incremental cost calculation, this occurs only under restrictive conditions. In reality, several situations exist that violate this condition. One such situation is early treatment termination, where withdrawal from the study intervention occurs due to adverse events, subject request, physician discretion, or death. This can result in a significant difference in the treatment (and observational) period between the arms. Additionally, unanticipated clinical events or unevenly distributed adverse events between treatment arms can lead to differences in disutility and data collection costs.

Moreover, protocol-driven activities can influence the absolute and relative risk reduction of the intervention compared to the control, even when protocol-driven activities are the same in both arms. This can result in the same costs but different patient utilities. For example, higher physician motivation in both arms can amplify the treatment effect, while a lack of motivation in both arms can diminish the treatment effect (in the sense of an interaction effect). Conversely, the same adherence measure may lead to a smaller incremental treatment effect if the control arm shows a larger relative improvement due to adherence measures (e.g., in the case of vitamin K antagonists) or if protocol-driven activities improve treatment of unrelated diseases or concomitant treatments in both arms, thus reducing the relative effectiveness of the investigational compound. Notably, since adherence is generally lower in real-world settings, under certain conditions, a new drug might show a greater additional health benefit than observed in the trial, contrary to conventional expectations [8].

The transferability of incremental costs and utility to the real world is also influenced by other differences between the trial setting and the real world, such as scale economies, capacity utilization, and hospital types [8]. However, these aspects are not the focus of this study.

If protocol-driven costs cannot be assumed to cancel out due to the conditions mentioned earlier, they need to be included in the ICER unless the cost and utility impact of certain protocol-driven activities can be separated [8]. However, the latter condition only applies to resources aimed at improving patient adherence under certain circumstances [8] and still requires the availability of a detailed account of protocol-driven activities. If costs to improve patient adherence cannot be separated, then all protocol-driven costs need to be included. In other words, although phase III trial costs are borne by the manufacturer, the trial resources contribute to patient utility demonstrated in the trial and would thus need to be covered by health insurance to achieve the same utility gain in the real world. Therefore, phase III trial costs are relevant from a payer perspective.

A comprehensive understanding of protocol-driven activities is crucial for accurately calculating protocol-driven costs. If a detailed account of these activities is unavailable, aggregated information on protocol-driven costs can be obtained from phase III trial costs. However, this approach necessitates identifying the portion of phase III trial costs that are protocol-driven. It is challenging to categorically reject any category of phase III trial costs mentioned by Sertkaya et al. [17, 18] as not being protocol-driven because all categories can at least partially have positive direct or indirect impacts on patient utility. For instance, administrative staffing costs may indirectly affect patient utility through better trial site management. Similarly, the need to prepare reports for institutional review boards may provide an incentive to improve patient care.

However, not all phase III trial costs can be deemed protocol-driven. For example, clinical staff time spent on general administrative duties that are not specific to the trial, such as completing routine paperwork, does not directly influence clinical outcomes or patient utility and therefore may not be considered protocol-driven. Another example is the time clinical staff spend administering a drug, which may also be required in the real world. Thus, including all phase III trial costs in the ICER and adding them to costs incurred in the real world could result in overestimation and double-counting. This holds from both societal and payer perspectives. Specifically, from a payer perspective, double-counting can occur in relation to activities that are already covered by health insurance in real-world applications. Therefore, to avoid double-counting from a payer perspective, this study includes all phase III trial costs that positively impact patient utility (because the insurer seeks to obtain and reimburse the utility gain demonstrated in the trial) but excludes phase III protocol-driven costs assumed to be covered by health insurance in a real-world application.

From a societal perspective, valuing resources based on true opportunity costs is an appropriate method [1]. This requires considering capital costs.

### Methodology

To capture the direct and indirect impact of phase III trial costs on patient utility, the analysis begins by excluding costs that are unrelated to patient utility from the overall phase III trial costs. Next, the analysis calculates the per-patient phase III trial cost (out-of-pocket) by dividing the total phase III trial cost by the number of patients enrolled. This cost is then divided by the per-patient trial participation period. However, the phase III trial length, which begins before any patients are enrolled and extends beyond the last patient’s follow-up, is not used because the denominator of the ICER refers to the health benefits generated during the phase of trial participation.

In a low-cost scenario, the time from the first visit after initial contact to the start of the trial is included, as protocol-driven activities begin before official trial enrollment and can influence patient utility. For example, the time taken by patients to review trial information, discuss it with their healthcare provider or trial staff, and make an informed decision is considered. Potential participants are also screened to determine if they meet the eligibility criteria specified in the trial protocol. The screening process typically involves medical assessments, laboratory tests, and sometimes genetic testing or imaging studies. Factors such as a washout period for certain medications or certain baseline measurements, can extend the time between obtaining consent and enrollment.

Finally, I multiply the estimated cost per unit of time by the additional trial-based observational period resulting from differential early treatment termination. In cases where a drug significantly affects mortality, additional survival time is used instead, even if the study treatment is not continued during the added survival time. This is because protocol-driven costs are incurred independently of ongoing treatment. For the period of “normal” life-years, it is assumed that the costs of phase III clinical trials cancel out due to the lack of published information on differences in protocol-driven activities. From a societal perspective, I apply the ratio of capitalized to out-of-pocket phase III costs as an additional multiplier.

### Data

While company-specific data on the trial and indication in question would be most appropriate for the analysis, they are rarely available for confidentiality reasons. Therefore, this study uses industry averages across indications and companies. There is considerable variation in reported average clinical trial costs and the average number of trial participants in the literature. Median estimates of trial costs range from US-$19.0 million [14] to US-$200 million [4] based on an ad-hoc literature review. Estimates by EvaluatePharma [7] and Sertkaya et al. [18], which are US-$127 million (median) and US-$20 million, respectively, fall in between. It is important to note that these estimates do not include capital costs and costs of failures. Additionally, the estimates pertain to a single phase III trial, whereas regulatory agencies (e.g., the European Medicines Agency) usually require at least two successful phase III trials. However, the costs of failures and confirmatory trials are not considered in this analysis, as they do not directly contribute to patient utility. Nonetheless, confirmatory trials may still reduce uncertainty regarding the added benefit, potentially leading to a utility gain.

Regarding trial size and duration, the study by DiMasi et al. [4] lacks information on mean and average trial size. Therefore, the study by EvaluatePharma [7] was used as an upper bound of clinical trial costs. According to the latter, the median estimates for orphan and non-orphan drugs are $99 million and $150 million, respectively, with an average cost across all trials of $163 million. The study includes “all new drug products entering phase III” from January 1, 2000, until 2015. Trial costs are higher than those reported by Moore et al. [14], which aligns with the smaller share (33%) of orphan-drug trials in EvaluatePharma’s study [7], as orphan-drug trials tend to have lower costs. It is worth noting that EvaluatePharma’s analysis does not specify the type of costs included (e.g., drug manufacturing costs).

The study by Moore et al. [14], which serves as a lower bound, includes pivotal trials for 59 new therapeutic entities approved in 2015/16 by the United States Food and Drug Administration, encompassing orphan drugs (46%) and biological drugs (31%). Notably, there is considerable variation in trial costs based on the therapeutic area, ranging from $9 million for “other” diseases to $157 million for cardiovascular diseases. The authors acknowledge that their estimates do not include costs borne by the sponsor, such as drug manufacturing or supervision of the contract research organization. Consequently, their study may have underestimated true costs.

In phase III clinical trials, a substantial portion of the costs directly impact patient utility due to the extended duration and intensive nature of these trials. These costs encompass direct medical expenses for treatment administration, diagnostic tests, and follow-up visits, as well as patient care costs for managing adverse effects and hospitalizations. In contrast, other costs such as patient recruitment and retention, some Clinical Research Associate (CRA) tasks, site recruitment and retention, administrative staff, site monitoring, data collection/management/analysis, Institutional Review Board (IRB) approvals/amendments, and Source Data Verification (SDV) are essential for the administrative and operational aspects of the trial but do not directly influence patient care or outcomes. While these costs indirectly benefit patient utility by ensuring valid and safe outcomes from the trial, they are not directly related to patient utility.

It is estimated that direct patient-related costs constitute 30–70% of the total trial costs. The lower end of this range (30%) represents trials with limited patient interaction, while the higher end (70%) reflects trials with extensive patient involvement. The higher estimate (70%) is applied in the higher bound scenario, indicating trials with significant patient engagement and interaction, while the lower estimate (30%) is used in the lower bound scenario, indicating trials with limited patient contact.

Regarding trial duration, EvaluatePharma [7] reported a median length of 2.88 years, although it is unclear whether this duration refers to the per-patient participation period or the overall trial duration (which begins before any patients are enrolled and extends beyond the last patient’s follow-up). Moore et al. [14] did not report an average or median length but stated that 64.5% of trials had a duration of 26 weeks or fewer, indicating that the median duration was less than 26 weeks. Most likely, in this case the reported duration refers to the per-patient participation period. In the low-cost scenario, a 4-week period was used to account for protocol-driven activities starting before trial enrollment. A 4-week pre-enrollment period positively can impact patient utility by conducting comprehensive screenings, managing potential health issues, and providing patient education, thereby optimizing patient readiness and engagement in the trial.

Regarding trial size, the analysis by Moore et al. [14] reported a median number of 488 patients. A similar estimate of 347 phase III trial patients was provided by Martin et al. [13]. In contrast, EvaluatePharma [7] reported a considerably higher median number of 921 patients (with an average of 2633).

To determine the costs from a societal perspective, the analysis considered the ratio of capitalized phase III trial cost (66.4 million) to out-of-pocket phase III trial cost (54.0 million) per investigational compound reported by DiMasi et al. [4] (66.4/54 = 1.23). Both costs account for the probability of failure in phase I and II trials.

## Results

The per-patient trial costs, calculated by dividing the phase III trial costs by the number of patients enrolled, range from approximately $17,520 (based on Moore et al. [14]) to $61,907 (based on EvaluatePharma [7]). Hence, the variation in per-patient trial costs is smaller than that of total trial costs. These costs were then multiplied by the percentage of direct patient-related costs, resulting in the values shown in Step 1 of Table 1.

**Table 1:** Calculation of the incremental per-patient phase III trial cost from the perspective of a payer (step 3) and society (step 4). All costs are in US dollars

Step		Lower bound	Upper bound	Average
1	Calculation of the per-patient phase III trial cost	5,256	43,335	24,295
2	Normalization to a one-year period	9,111	86,669	47,890
3	Adjustment to a three-month period	2,278	21,667	11,972
4	Inclusion of capital costs	5,078	48,310	26,694

To calculate per-patient trial costs over one year, I used an estimate of 0.5 years based on Moore et al. [14], who reported a median per-patient participation period of less than 26 weeks; in the lower bound scenario, a 4-week period was added as per the Methods section. This results in a rather high estimate of approximately $48,000 per patient over a one-year trial period (averaged over the two estimates for per-patient trial costs). Therefore, a treatment that prolongs life, for example, by three months would imply an average phase III cost of $12,000 from a payer perspective that needs to be added to the numerator of the ICER. Refer to Table 1 for estimates in the low-cost and high-cost scenarios. Using the ratio of capitalized to out-of-pocket expected phase III trial cost, which is 1.23, I obtain $27,000 (12,000 + 12,000 × 1.23) per-patient societal costs over a three-month trial period.

## Discussion

This study argues for the inclusion of phase III trial costs in the ICER when protocol-driven costs do not cancel out, such as when the investigational compound leads to longer survival. The only exception among the various protocol-driven cost components are the costs to improve patient adherence, which may be excludable under certain conditions but require a simultaneous adjustment of clinical outcomes [8].

This study demonstrates that the impact of phase III trial costs on the ICER is not negligible even when the duration of treatment is extended only within a range of weeks. Phase III trial costs can be of similar magnitude as medication costs. The inclusion of phase III trial costs becomes most significant for decision-makers when the ICER is close to the threshold willingness to pay. However, it is important to note that adding phase III trial costs to the numerator of a conventionally calculated ICER results in double-counting. This is because conventional calculations already consider the portion of phase III trial costs incurred in a standard real-world application. Therefore, when including the estimate of the phase III trial cost of $27,000 (from a societal perspective) in the ICER, the costs of protocol-driven activities covered by health insurance in the real world would need to be excluded (see the Methods section). To account for the latter, a useful approach, at least in some jurisdictions, is to consult the prescribing information of a drug, which specifies the required or recommended drug-related services such as drug application, counseling, monitoring, and testing. Ultimately, for a precise analysis of phase III trial costs specific to a particular drug, company-specific data should be used during the differential treatment and observation period, rather than relying on the averages applied in the example provided.

Importantly, from a societal perspective, when including only the marginal costs of producing and distributing the drug only (according to Garrison et al. [10]), adding phase III trial costs clearly does not result in double-counting of drug costs. Conversely, if the societal perspective also considers dynamic efficiency, which aims to promote innovation, double-counting would occur, as drug prices would then need to reflect the opportunity cost of R&D. From a payer perspective, double-counting does not occur under a value-based pricing (VBP) scheme of drugs, where the “gold standard” for VBP calculations is the ratio of costs to quality-adjusted life-years [15]. This is because, under VBP, the drug price is exogenous to R&D costs and is exclusively based on value by definition. From the payer perspective, value is independent of R&D costs unless the payer (government) invests in later-stage clinical research. In such cases, VBP with an adjustment for R&D costs is justified by subtracting the portion of the value that derives from government-funded R&D costs [15].

A CEA for the duration of a clinical trial can be conducted using primary data collection (referred to as “piggyback” economic evaluation) or decision modeling. Regardless of the approach, protocol-driven costs need to be added in both instances to account for the full impact of the intervention on patient utility.

Our analysis, which focuses on the added treatment period, assumes that during the parallel treatment in both arms, the costs of phase III trials cancel out. However, this may not be true based on the reasons outlined in the Methods section. Additionally, it assumes that the impact of protocol-driven activities on clinical outcomes is the same in both arms during parallel treatment (refer to the Methods section). However, the incremental utility of considering protocol-driven activities may not always be positive; the utility may be larger in the control arm.

Interestingly, this analysis indirectly addresses an ongoing debate among governments, payers, and manufacturers regarding the pricing of new drugs. VBP has become a new paradigm for pricing of new, innovative drugs, making cost-based or cost-plus pricing (which includes pricing based on the profit margin or the profit-to-cost ratio) comparatively less popular, at least in formal pricing exercises. One reason for the lack of interest in cost-based pricing is that it does not incentivize efficient R&D. Nonetheless, decision-makers may still feel that information on the cost of R&D is necessary for transparency and fairer funding decision-making [11]. Moreover, cost considerations remain relevant particularly for the pricing of orphan drugs, as manufacturers often justify high prices by the high R&D expenses incurred to bring these drugs to the market.

However, the cost of bringing a new drug to market is mostly non-transparent [19] and it remains one of the greatest controversies within the pharmaceutical industry. The analysis conducted repeatedly at the Tufts Center for the Study of Drug Development over 25 years, authored by DiMasi, Hansen, and Grabowski, is a well-known and often-cited source. Their most recent estimate (2016) suggests a total cost of $2.6 billion in 2013 dollars to develop a single drug and gain marketing approval. It is important to note that this estimate includes capital costs (i.e., the opportunity cost of developing these drugs) and the costs of failures, which are the major drivers of this estimate. However, the estimate has faced criticism for various reasons, including unrepresentativeness/selection bias (as companies could choose to participate [14], potentially biased data (self-reported by companies), failure to adjust for tax savings for companies, consideration of capital costs, and overestimation of the costs and duration of clinical trials [12]. Light and Warburton [12] concluded that DiMasi et al.’s 2003 estimate overestimated R&D costs by factor 18. Similarly, Prasad and Mailankody [16] suggested a lower estimate of $757 million (including opportunity costs) based on an analysis of cancer drugs produced by companies with no other drugs in the market. However, DiMasi [5] commented on the study by Prasad and Mailankody [16] that the included companies had a higher success rate than the average.

These discrepancies, along with the considerable variation reported in the literature regarding average phase III trial costs and the average number of trial participants discussed earlier, indicate a disincentive for pharmaceutical companies to disclose full information due to the potential competitive disadvantage it may create.

Based on the argument that manufacturers should include costs of protocol-driven activities in cost-effectiveness analyses alongside clinical trials, this study provides a framework for accurate reporting of phase III trial costs. On the one hand, pharmaceutical companies have an incentive to improve the ICER of their products by excluding protocol-driven costs while incorporating the utility gain from protocol-driven activities. On the other hand, there is an incentive for them to overstate the costs of phase III clinical trials under cost-plus pricing. Thus, an inherent conflict arises in the perspective of phase III trial costs between value-based and cost-based pricing. Requiring the inclusion of trial costs can result in a significant increase in the ICER and a corresponding decrease in the value-based drug price. Simultaneously, it raises the cost-plus drug price. By mandating the inclusion of trial-based costs in the ICER, current incentives to overstate R&D/phase III trial costs and understate protocol-driven costs would be mitigated, at least in situations where pricing is based both on value and costs. The drawback of cost-plus pricing, which is the lack of incentive to improve R&D efficiency, would also be alleviated.

In conclusion, this article establishes a connection between two cost components (phase III trial costs and incremental costs) that have historically been considered in isolation to date. This connection arises from the necessity to include the costs of protocol-driven activities in the ICER from both a societal and payer perspective for the sake of consistency. As an unintended consequence, implementing this approach aids in revealing the true R&D cost of pharmaceuticals without imposing a requirement for manufacturers to openly disclose them.

## Data Availability

Data is provided within the manuscript.
